# How people used ochre at Rose Cottage Cave, South Africa: Sixty thousand years of evidence from the Middle Stone Age

**DOI:** 10.1371/journal.pone.0176317

**Published:** 2017-04-26

**Authors:** Tammy Hodgskiss, Lyn Wadley

**Affiliations:** Evolutionary Studies Institute, University of the Witwatersrand, Johannesburg, South Africa; Universidade do Algarve, PORTUGAL

## Abstract

We describe colour, hardness, grain size, geological type and surface modifications of ochre pieces excavated, first by Malan and later by Harper, from the Middle Stone Age (MSA) of Rose Cottage Cave, 96, 000 to 30, 000 years ago. Soft, bright-red shales are abundant, and most ochre has clayey or silty grain sizes. The post-Howiesons Poort layers contain the most ochre pieces, but the Howiesons Poort layers have the highest frequency of ochre per sediment volume. The pre-Howiesons Poort layers have the highest utilization rate. Use-traces include rubbing, grinding, combined grinding and rubbing, and rare instances of scoring. The processing techniques are proxies for ochre use. Rubbing transfers red ochre powder directly onto soft surfaces, such as human skin, or animal hide. This is appropriate when skin colouring and marking or skin protection (for example from sun, insects or bacteria) is the purpose. Grinding produces ochre powder that can be used for a variety of tasks. It can be mixed with water or other products to create paint, cosmetics or adhesives. Multiple uses of ochre powder and ochre pieces are therefore implied at Rose Cottage and changes through time are apparent.

## Introduction and background

Ochre is an informal term used to group a range of ferruginous rocks containing iron oxide like hematite (α-Fe2O3), or hydrated iron oxyhydroxide like goethite (α-FeOOH) [1]. Broadly speaking, the colour red correlates with hematite-rich ochre, while yellow correlates with goethite [2]. We use the term ‘ochre’ to describe these rocks which leave a coloured streak, rather than ‘pigment’ or ‘colouring material’ to avoid assumptions on why and how the material was used in the past (i.e. Watts [3]). Ochre has a long record of collection and use. In both Africa and Europe, ochre use predates the evolution of *Homo sapiens* (for Africa see [4–9]; and for Europe see, for example [10, 11]), though [12] suggests that routine exploitation of red ochre use is a “species-defining trait” of *Homo sapiens*. In the southern African Middle Stone Age (MSA), ochre is thought to have been collected and used by 500,000 years ago at Kathu Pan [9], and worked ochre is present in the 164,000 year old layers of Pinnacle Point Cave 13B, on the Cape coast [13, 14] as well as in the late MIS 6 sediments at Wonderkrater, a Limpopo spring site [15]. In more recent MSA assemblages, ochre is abundant and there is greater evidence for a variety of uses for the product. Ochre was worked in Apollo 11 [16–18], Blombos Cave [19], Klipdrift [20], Border Cave [21], Hoedjiespunt [22], Klasies River [23–25], Die Kelders [26], Hollow Rock Shelter [27], Diepkloof [28, 29], Sibudu [30], Zombepata [31] and many other sites (see [3, 32]). It is a regular feature in all Cape west coast MSA shell middens [33] and most Late Pleistocene caves across southern Africa (Watts [3, 34]).

Today there are fewer polarizations than there were ten years ago between archaeological interpretations of the use of ochre in the past. The potential for multiple uses has been well established through experiments (for example, [35–44]), and ochre is accepted as having potentially been used as an insect repellent, sun screen, body paint, hide tanning agent, anti-bacterial agent, and colorant for a range of artefacts and ornaments. Practical uses for ochre do not necessarily contradict the potential for ochre’s symbolic role. It is less simple to demonstrate a multiplicity of ochre use from archaeological evidence, but to some extent use is implied by finds of ochre-loaded adhesives [45–48], ochre in burials [49, 50], ochre on bone tools that may have been awls used for piercing hides [19], ochre-based paint [51, 52], engraved ochre [53–56] and ochre in (perforated) shells [57, 58] that may have been deliberately coloured or worn suspended on painted bodies or ochre-rubbed hides. Some of these activities seem time restricted and this may have resulted in changes in the types of ochre selected.

Our new study of the ochre assemblage from Rose Cottage Cave, eastern Free State, South Africa, adds to knowledge of potential ochre use during the MSA. The site is important not only for its long cultural sequence, but also because it is inland and far from Cape coastal sites that have received greater attention.

A preliminary study of the ochre was made by Watts [3]. Here we reanalyse the Rose Cottage MSA ochre from Malan’s excavation and add an analysis of the unpublished specimens from Harper’s excavations [59, 60]. The long sequence from Rose Cottage has been dated since the Malan and Harper excavations [61–63] and the MSA has ages between about 96,000 years and 30,000 years ago (Table 1). The sequence contains a rich Howiesons Poort (HP) industry as well as earlier and later MSA assemblages [60, 64–66]. Malan never published his Rose Cottage excavations, other than in a short note [67], but Wadley and Harper [64] conducted a typological analysis of the lithics he recovered. This analysis demonstrated that Malan did not keep all artefacts, but selected those he thought important. This must be borne in mind when comparing the quantities of ochre from Rose Cottage with those from more recently excavated sites with larger recovery rates.

**Table 1:** Rose Cottage Cave Optically Stimulated Luminescence (OSL) and Thermo-luminescence (TL) ages for MSA layers at Rose Cottage Cave.

Technocomplex	Age (ka) ^a^	Layer	Method ^b^
**Pre-Howiesons Poort**	95.9 ± 6.6	LEN	OSL
	72.5 ± 6.8	LEN	TL
	76.3 ± 14.8	LEN	TL
	68.4 ± 8.3	LEN	TL
	64.5 ± 6.6	LEN	TL
	71.4 ± 4.2	KUA	OSL
**Howiesons Poort**	48.9 ± 5.3*	EMD	TL
	65.0 ± 3.0	EMD	OSL single grain
	68.7 ± 2.7	EMC	OSL
	63.2 ± 2.3	ENG	OSL single grain
	63.0 ± 2.3	BER	OSL single grain
	60.4 ± 4.6	BER	TL
	56.3 ± 4.5	BER	TL
	54.0 ± 4.4	BER	OSL
	62.5 ± 2.9	ETH	OSL
	41.7 ± 3.7*	ETH	TL
	58.6 ± 6.6	SUZ	TL
**Post-Howiesons Poort**	50.5 ± 4.6	BYR	TL
	61.8 ± 2.8	ANN	OSL
	56.0 ± 2.6	LIN	OSL single grain
	59.4 ± 4.5	CLI	OSL
	49.4 ± 10.1	CLI	TL
	47.1 ± 10.2	THO	TL
	35.8 ± 2.4	LYN	OSL
	34.8 ± 2.2	LYN	OSL
	29.8 ± 1.6	DC	OSL
	27.6 ± 2.3	RU	OSL
	31.7 ± 1.8	G	OSL

^a^Incoherent ages that are probably the result of bleached lithics are marked *

^b^OSL dates were obtained by [63], OSL single grain by [62] and TL by [61].

## Rose Cottage Cave (RCC)

**Fig 1:**
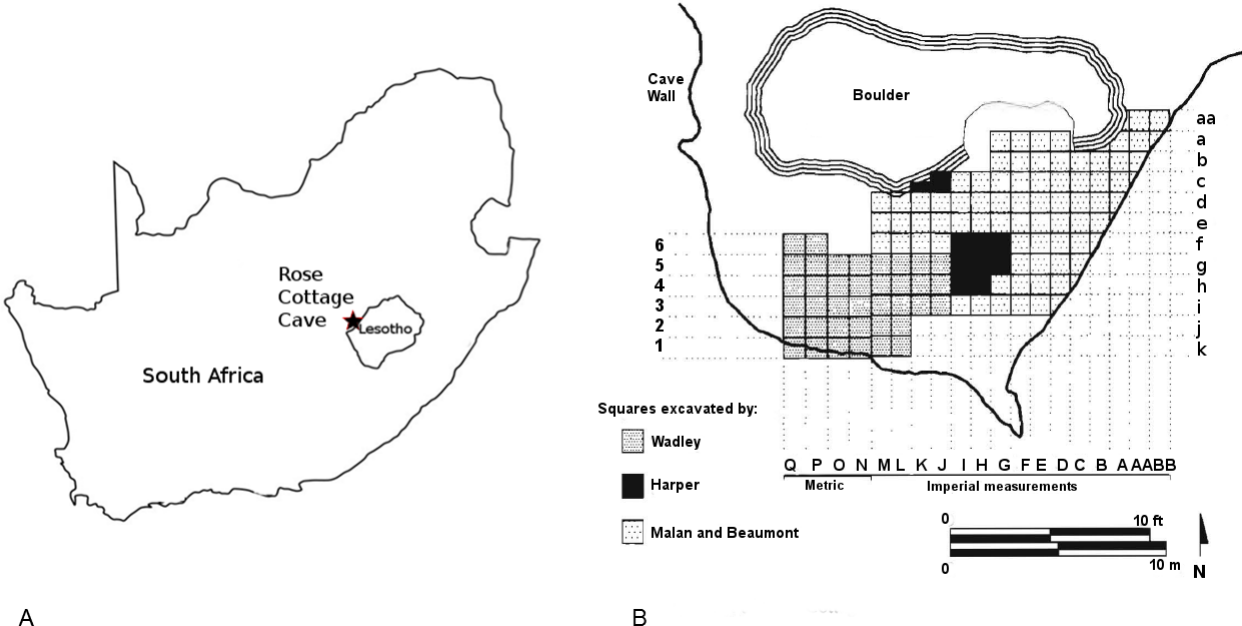
Rose Cottage Cave. A. Location of Rose Cottage Cave in South Africa. B. Plan of Rose Cottage Cave showing the location of squares excavated by Wadley, Harper, Malan and Beaumont (modified from [70]).

Rose Cottage is located near the Caledon River and close to Ladybrand in the eastern Free State, South Africa (Fig 1A). It is on the northern slopes of the Platberg and is about 20 m long and 10 m wide and is protected by a great boulder at the front of the cave. The cave sediments, more than 6 m deep in parts of the cave, formed mainly through the weathering of its sandstone roof and walls, and through materials washed in by springs [68, 69]. No ochre source occurs in or near the cave.

**Fig 2:**
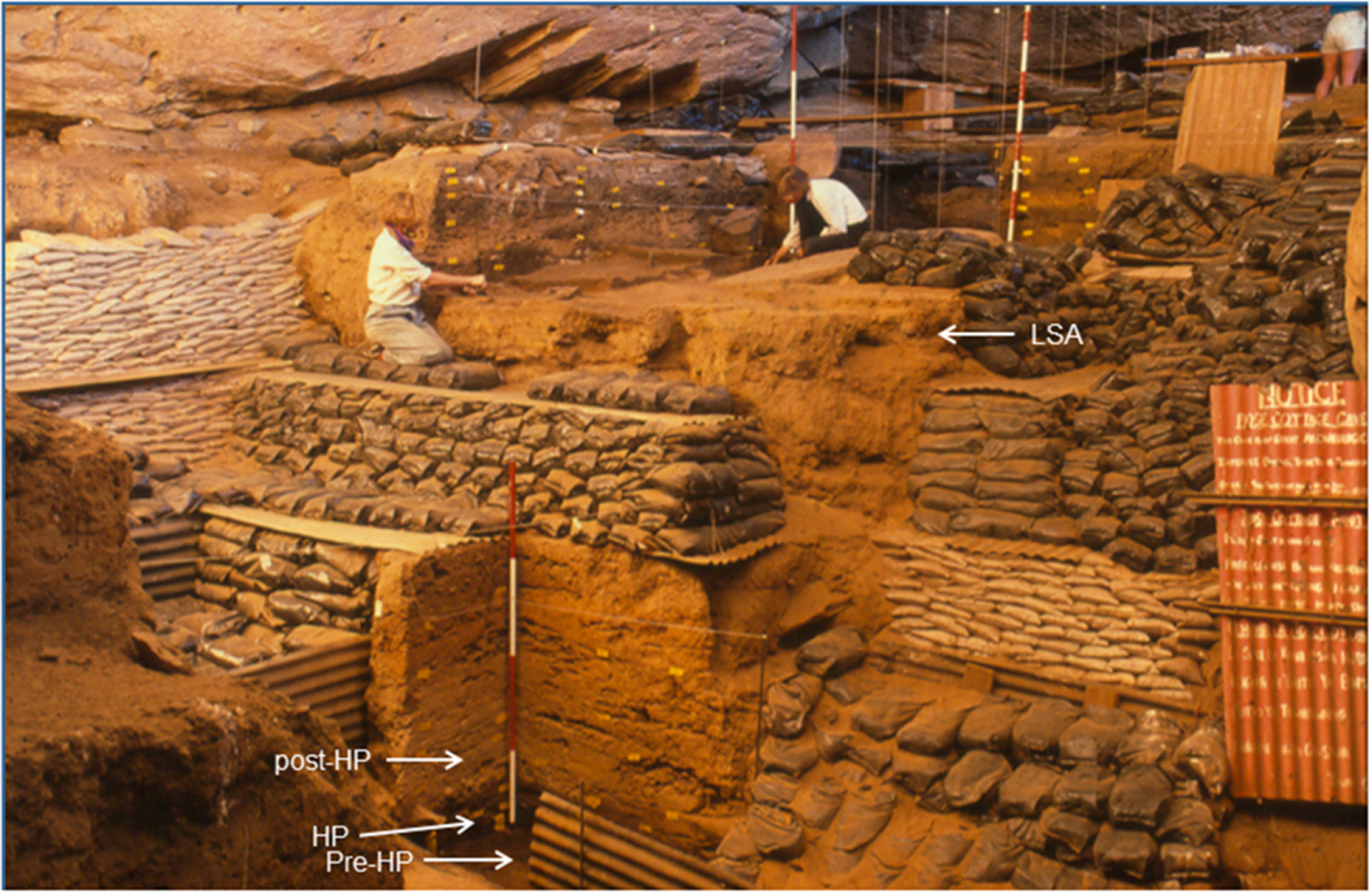
Excavations at Rose Cottage Cave. Wadley LSA excavations at the top; Harper excavations marked pre-HP, HP and post-HP (see also Fig 3). The post-HP sequence extends to the top of the measuring rod. Malan’s excavations are in the foreground and under the bags to the left. The Beaumont excavations were under the bags to the right. The bags and boards were part of the rehabilitation by Wadley.

**Fig 3:**
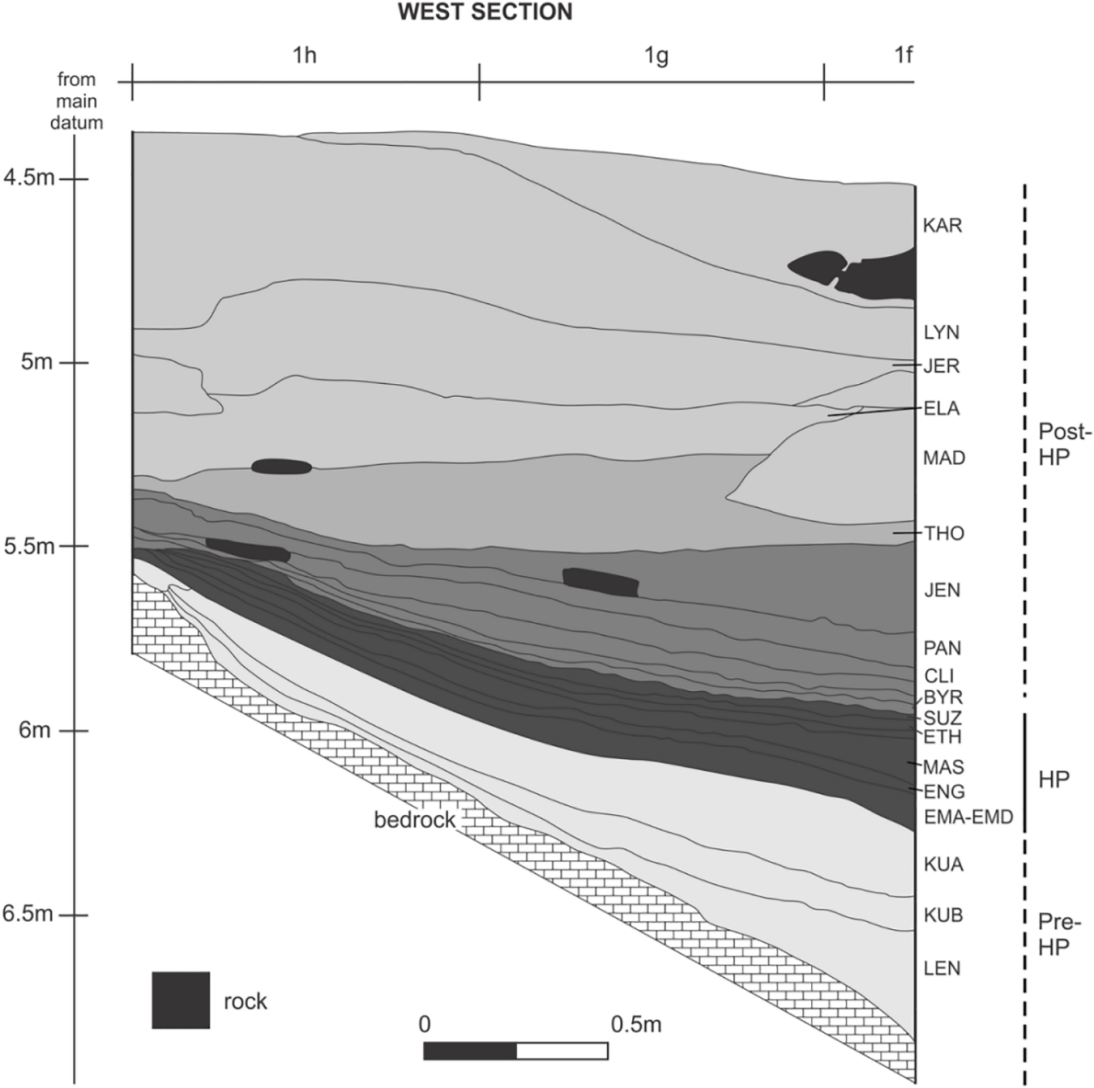
Stratigraphy of the west wall of the Harper excavation into MSA sediments at Rose Cottage Cave. A long sequence of LSA sediments is missing above KAR because the squares were excavated to that depth by Malan. The pre-HP, HP and post-HP associations are marked on the right.

Rose Cottage was excavated by Berry D. Malan 1943–1946, by Peter B. Beaumont in 1962, by Lyn Wadley from 1987 to 1997, and Philip Harper, under Wadley’s supervision, in 1989. The Malan and Beaumont excavations yielded both LSA and MSA material. Malan excavated in 3 inch spits, but some spits were 6 or 9 inches deep depending on the stratigraphy and natural homogeneity in the sediment. The spits blurred assemblage boundaries and it is therefore difficult to correlate the assemblages from the various excavations. Malan excavated a large area of the cave in the upper levels (Fig 1B), but only five squares (measured in yards) into the deepest MSA layers (Fig 2). [64] attempted to match Malan’s spits and the cultural sequence; here we shall use the groups of spits and cultural sequence that they proposed. Harper excavated nine squares of undisturbed MSA sediment (Fig 1B). His layers followed the natural strata (Fig 3). Wadley excavated a large area, but concentrated on the LSA occupations and terminated her excavation in the upper MSA layers (Fig 2). In contrast, Harper’s excavations were exclusively into MSA sediments exposed by the Malan and Beaumont excavations (Fig 3). Our study deals only with the MSA ochre assemblage, therefore the LSA excavations and data are not discussed further.

The MSA ages are derived from Optically Stimulated Luminescence (OSL) on sediments and Thermo-luminescence (TL) on burnt lithics: the ages represent at least 60, 000 years of intermittent MSA occupations at the site, beginning at about 96,000 years ago and ending at about 30,000 years ago [61–63]. The MSA lithic assemblages include a recognisable HP, within which temporal variation is visible, but the earlier and later ones do not resemble those from other published sequences and were therefore called pre-HP, and post-HP by [64].

Wadley and Harper performed extensive lithic analyses [59, 60, 64, 70–72]. The assemblages mostly comprise fine-grained opalines from the Drakensberg basalts of the Clarens Formation, many of which would have been washed by mountain streams into the Caledon River, about 10km from the cave [71, 73]. The HP layers are distinguished by the presence of many backed tools (segments [crescents, lunates], backed blades and obliquely backed blades [truncations]), bladelets and thin blades [60, 64]. Wadley and Harper [64] documented erratic frequencies of lithic chips and chunks in the Malan collection, possibly because he did not systematically keep all pieces. Irregular collection practice is also likely to affect the frequencies of ochre pieces in his assemblage, particularly those of small pieces with no clear evidence of use wear.

## Previous studies of the Rose Cottage Cave ochre

### The ochre collection

The ochre from the various Rose Cottage excavations is curated in Archaeology Department Origins Centre basement, University of the Witwatersrand, Johannesburg under the catalogue number “2927AB5 Rose Cottage Cave”. Ian Watts [3, 32] studied the Malan MSA and Wadley transitional MSA and LSA ochre collections, but not the Harper collection. Watts’s investigation of the Malan ochre incorporated 84 definite and 25 ‘possible’ ochre pieces. He concluded that the highest ratio of ochre pieces was in the oldest levels (associated with lithics called MSA2b, Volman’s classification [74]) while the lowest ratio was in the post-HP layers. He saw that high quality crimson ochre and hematite are in larger proportions before and after the HP (colour tests involved streak analyses that were then described using Munsell notations). He concluded that scored ochre from the oldest MSA layers was non-functional scoring, i.e. not a result of powder-production, but perhaps engraving. His study provided valuable descriptive details of the physical nature of the ochre pieces.

Harper [59] recorded 382 ochre pieces and found that colours ranged from rich reds to yellows, with a range of sizes (from 1–4 cm) and with some grinding and scoring. Importantly, the temporal patterns he recognized match those that Watts saw in the Malan collection–that is, greater ochre (to lithic) frequencies in the earlier MSA and the post-HP layers and a drop in ochre frequency during the HP.

### Ochre residues on Rose Cottage Cave MSA lithics

Twenty-eight backed tools with ochre residues were found among the 68,000 to 60,000 year old HP lithics from the Harper excavation [45]. Ochre and plant residues are mostly concentrated on or close to the backed edges (n = 27) of the tools, although some ochre residues occur elsewhere on the tools (n = 15 on shafts and n = 9 on cutting edges). The positions of the ochre, plant fibre, plant tissue and starchy deposits are statistically significant and are unlikely to have happened by chance [45]. It is most probable that the substances were adhesives used to haft the tools.

Harper [59] reports a post-HP palette with thick colouring material (ochre powder) on it. However, there is no further description of this stone that came from the Malan excavation. Both Malan and Beaumont recorded grindstones and hammer stones [3], but none had ochre residues on their surfaces. There are reports of four grindstones/hammerstones in square Fb in the front of the inner cavern (in the mixed pre-HP and HP layers) [3: 831]. One grindstone with ochre residues was found in the Wadley excavation of the final MSA [75] and there is one sandstone grindstone in the Harper excavation of a Howiesons Poort layer (square Hf, layer MAS).

## Materials and methods

The Malan ochre assemblage analyzed here is from squares Da-Dd, Ea-Ef, Fb-Fg and Ga-Gc (Fig 1B). Unfortunately, the ochre pieces from these 1940s excavations were packed in bags with the lithic assemblages and many have post-depositional damage. All ochre pieces from the Harper excavation (squares Gf, Gg, Hf, Hg, Hh, If, Ig, Ih, Jc, Kc) were successfully located and are analyzed here. Malan excavated in spits and Harper excavated stratigraphically, so it is difficult to combine or compare the assemblages. Nonetheless, we have, unless otherwise stated, grouped together the Malan and Harper collections throughout this paper. We do so by placing the ochre in broad categories pre-HP, HP and post-HP. Another category, Mixed pre-HP/HP, was created to accommodate Malan collections from spits that cross-cut the two techno-complexes.

Each ochre piece was measured and given an individual code. Refitting was attempted within each quadrant (by stratigraphic layer) and each refit was counted as one piece. All pieces >8 mm from the Malan and Harper collections were individually numbered and stored in separate plastic bags. The geological properties of pieces < 8 mm cannot be reliably identified and so these pieces were excluded. Each piece > 8 mm was then macro- and microscopically examined for use-traces using an Olympus SZ61 Zoom Stereo microscope, with a WHS-10x FN22 eyepiece (0.67 to 45x magnification) and photographed using a Panasonic Lumix Camera and an Olympus D212 microscope camera.

All pieces (utilized, unutilized and possibly utilized) with a flat surface larger than 8 mm were analyzed using a Thermo Scientific Niton XL3t 950 portable XRF analyzer. The analyzer is fitted with a GOLDD+ drift detector and a miniaturized x-ray tube with a 50 kV excitation source. Each piece was tested for 180 seconds. These preliminary tests were performed to obtain semi-quantitative data on the inorganic elements present in the ochre pieces.

### Physical properties

Using established methods and criteria for the examination of MSA ochre [3, 30, 38, 76–78], the physical properties were recorded for each piece and categorized accordingly (Table 2, for further details on specific methods used, see [30]). The following physical properties were identified:

1. *Geological type and mica inclusions*. Pieces were assigned to geological categories. Broad classification categories were used because pieces are heterogeneous and identification to a more specific level can be unreliable. Shale is recognised by the presence of fissile laminations. Siltstone, mudstone and sandstone are distinguished from each other by grain size. Snuffbox shale is nonfissile and is created during the formation of the rock when pyrite (FeS_2_) fills the original bedding planes within the shale, creating ‘boxes’ of hard ironstone around soft shale pockets [79]. ‘Iron oxide’ is a general grouping for dense, hard materials such as chemically altered shale and hematite. Pieces that could not be definitively identified or were a combination of forms were placed into a separate category, ‘other’. The presence of mica inclusions is noted. The surface of mica-rich pieces display small sparkling particles.
2. *Grain Size*. Pieces were assigned to approximate grain size or textural groupings: clayey (diameter <4 μm), silty (diameter 4–50 μm) and sandy (diameter ~50 μm– 2 mm) [80]. Often pieces have a combination of grain sizes, making a sliding scale necessary: clayey, clayey/silty, silty, silty/sandy and sandy.
3. *Hardness*. Hardness values were assigned to each piece using Mohs’ hardness scale. Values were then grouped to aid analysis: soft is Mohs 2 and below, medium is Mohs 3 and 4, and hard is Mohs 5 and above.
4. *Colour*. The surface colour of ochre pieces may be different from the colour of the powder it produces. To obtain an indication of the colour of the powder most likely to be created, streak tests were performed. This was done by rubbing the ochre piece against a white, unglazed porcelain tile. Streaks were then given Munsell Colour Chart codes. Munsell Colours were grouped into colour groups based on a polythetic divisive classificatory program, POLYDIV [81].

**Table 2:** Recorded physical properties of Rose Cottage Cave ochre and their identifying features.

Ochre attributes	Identifying features
Geological type	Shale, mudstone, siltstone, sandstone, snuffbox shale, iron oxide, other
Brilliance	Mica inclusions present or absent
Grain size	Texture: clayey, clayey/silty, silty, silty/sandy or sandy
Hardness	Soft (Mohs Scale 1 and 2), medium (Mohs 3 and 4) and hard (Mohs 5 and above)
Colour	Munsell colour readings on streaks. Colour groupings are made with a polythetic divisive classificatory program [81]

### Use-traces and wear

Pieces were examined for use-traces and then classified as utilized, possibly utilized and unutilized. Pieces with use-traces were further macro- and microscopically examined. Grooves are described by their orientation, placement, profile shape, termination type and the presence or absence of internal microstriations. Polish, metallic lustre, smoothing and external microstriations on the piece surface, surface shape and edge shapes are recorded. Post-depositional markings, such as scratches, scuffmarks and smoothing (occurring independently of polish, external microstriations and metallic lustre) are also noted. Almost 90% of the ochre pieces analysed here have some form of post-depositional damage. This damage is not restricted to the ochre; [82] reported a high incidence of metal scratches and modern contamination on the lithics she analysed. These effects would be especially pronounced for soft materials such as ochre. The post-depositional damage has made identification of the use-traces difficult.

Once all use-traces and surface features are identified, the activity responsible for the use-traces is assigned, based on use-trace combinations and positioning. Activities include–rubbing, grinding, scoring and combinations thereof. The use-types and activities that could be confidently recognized are discussed here. For more information on use-trace and activity identification protocol, terminology and descriptive criteria see [77, 78]. We now briefly describe the use-traces indicative of the activity categories.

1. *Rubbing*. Rubbing activities involve rubbing an ochre piece against a soft material, such as animal hide or human skin. The most common use-traces that form during this activity are smoothing, edge rounding, external microstriations and polish. Smoothing can also be caused by post-depositional processes. To ensure post-depositional markings are not confused with anthropogenic rubbing, the following indicators are sought: polish, external microstriations and metallic lustre. Such use-traces are likely to be removed during post-depositional processes. The hardness of the raw material is important for distinguishing between activity-related use-traces (grinding and rubbing) and post-depositional markings (smoothing). For example, the surface shape of medium and hard pieces is unlikely to change when rubbed against a soft material, but the surface shape of such pieces would change when ground, and then facets may form. Intentional rubbing of soft pieces could result in surface shape change, i.e. the creation of flat or faceted surfaces. However, soft pieces may look rubbed, but their smoothing may be post-depositional or from handling at time of use. To differentiate we look for additional features such as polish and metallic lustre to confirm rubbing actions. If this use-wear cannot to confidently identified as having been caused by rubbing, then the pieces are placed in the possibly utilized or unutilized categories.
2. *Grinding*. Use-traces from grinding ochre pieces against a hard surface appear as multiple, parallel grooves (striations) that usually reach the edges of both surfaces. The striations have unfrayed edges and will contain internal microstriations, unless they have been rubbed, smoothed or wet after grinding. Grinding causes surface and edge shape changes, commonly resulting in flat, faceted surfaces. Grinding can produce metallic lustre and polish around the striations, but this is not a consistent use-trace related to grinding (it may be determined by the mineralogy or hardness of the pieces).
3. *Scoring*. Scored incisions often do not reach the edges of the piece. These incisions usually contain microstriations, unless the piece has been rubbed or smoothed afterwards. The incisions can have frayed terminations implying that each incision was created with multiple strokes. Profile shapes and depth of the incisions vary greatly, depending on the tool type used and whether the incision was re-incised or not. Scoring is further classified as light or intensive. Intensive scoring causes multiple incisions that seem randomly placed. Light scoring gives the appearance of carefully placed incisions that have been re-incised (or engraved).

Pieces can also display evidence for more than one activity. Pieces with evidence of grinding and rubbing often have flat or faceted surfaces. Rubbing previously ground pieces causes widening, smoothing of the grinding striations, the removal of internal microstriations and can result in the removal of grinding striations. The hardness of the piece is also considered when designating activity categories, as this influences the use-traces that form (see above).

## Results

The Rose Cottage Cave Harper and Malan ochre assemblages comprise 618 pieces. Pieces ≤ 8 mm were excluded from detailed analysis, making the analysed assemblage 529 pieces with a total weight of 2.75 kg: 405 pieces in the Harper collection (1.28 kg) (we found some that went unrecognized by Harper) and 124 in the Malan collection (1.47 kg). There are 59 pieces that show signs of definite utilization (11.2% of the assemblage) and another 42 pieces with possible utilization. The ochre assemblage from the pre-HP layers has the highest proportion of pieces with utilization– 21.3% (Table 3). The post-HP and HP have considerably lower proportions of utilized ochre– 10.1% in the post-HP and 7.6% in the HP. The pieces from the ‘Mixed pre-HP and HP layers’ (n = 8) are excluded from the sample when determining variations between the technocomplexes.

**Table 3:** Temporal variations in unutilized, possibly utilized and utilized ochre frequencies in the MSA layers at Rose Cottage Cave (Malan and Harper collections).

Technocomplex	Total Ochre	Unutilized	Possibly Utilized	Utilized	% Utilized	Ochre piece density ^a^	Ochre weight density ^b^
Post-HP	297	241	26	30	10.1%	0.7	1.1
HP	144	121	12	11	7.6%	1.7	11.5
Pre-HP	80	61	2	17	21.3%	0.2	6.4

Densities are calculated with the Harper assemblage.

^**a**^Piece density = number of ochre pieces per 10 litres of sediment.

^**b**^Weight density = grams of ochre per 10 litres of sediment.

### Ochre volume density and spatial distribution

The lithic analysis of the squares that Harper excavated [59, 60] showed that lithic densities are at their highest in the HP (131.5 tools per ten litres of deposit removed); followed by the post-HP (27.91) and the lowest tool densities are present in the pre-HP (9.39). Ochre pieces are also at a higher volume density in the HP compared to the other industries (Table 3). Volume densities can only be calculated from the Harper excavation where a record was kept of excavated sediment.

However, it is possible to say something about the spatial patterns of ochre in the Malan excavation. The pre-HP ochre that has traces of both grinding and rubbing activities was almost entirely recovered from the eastern side of the cave (in Malan’s squares Gb, Gc, Fb and Fc; Fig 1B).

### Physical properties

#### Geological type

The utilized and unutilized assemblages have similar proportions of geological forms, with shales constituting the majority of both the assemblages, but featuring more prominently in the utilized assemblage (Table 4). Of note is the higher percentage of iron oxides in the utilized as opposed to the unutilized assemblage, particularly in the pre-HP. Siltstones, sandstones and snuffbox shales are most common in the unutilized assemblage. A higher percentage of shale is utilized in the post-HP (compared to the other industries), whereas the HP has an increase in utilized siltstone. Rose Cottage red ochre has a high percentage of Fe_2_O_3_ [36]. A geological sample of ochre from a rock shelter in the Ladybrand area (about 20 km from Rose Cottage) had a slightly lower Fe_2_O_3_ (25%) and higher SiO_2_ (63%) readings. This suggests that the occupants of Rose Cottage may deliberately have sourced and selected ochre with high Fe_2_O_3_ content. However, we suggest this cautiously because we have tested only a small sample of ochre.

**Table 4:** Analysis of the properties of the utilized and unutilized ochre pieces from the earlier MSA at Rose Cottage Cave.

		Post-HP (N = 271)		HP (N = 132)		Pre-HP (N = 78)		Total (N = 481)	
OCHRE PROPERTIES		Unutilized	Utilized	Unutilized	Utilized	Unutilized	Utilized	Unutilized	Utilized
		n = 241	n = 30	n = 121	n = 11	n = 61	n = 17	n = 423	n = 58
		% n	% n	% n	% n	% n	% n	% n	% n
Geological Form	Shale	48.5	76.7	58.7	54.5	42.6	64.7	50.6	69
	Mudstone	7.1	10	3.3	9.1	13.1	11.8	6.9	10.3
	Siltstone	10	3.3	9.9	18.2	4.9	-	9.2	5.2
	Sandstone	18.3	6.7	13.2	9.1	16.4	-	16.5	5.2
	Snuffbox Shale	11.6	-	7.4	-	21.3	-	11.8	-
	Iron Oxide	4.1	3.3	5	9.1	1.6	23.5	4	10.3
	Other	0.4	-	2.5	-	-	-	0.9	-
Grain Size	Clayey	39.4	66.7	52.1	45.5	54.1	35.3	45.2	53.4
	Clayey/Silty	7.1	6.7	3.3	9.1	6.6		5.9	6.9
	Silty	22.8	16.7	17.4	18.2	14.8	58.8	20.1	29.3
	Silty/Sandy	18.3	6.7	17.4	18.2	11.5	-	17	6.9
	Sandy	12.4	3.3	9.9	9.1	13.1	-	11.8	3.4
Hardness	Soft	55.2	63.3	71.9	54.5	67.2	70.6	61.7	63.8
	Medium	43.6	36.7	26.4	36.4	32.8	29.4	37.1	34.5
	Hard	1.2	-	1.7	9.1	-	-	1.2	1.7
Colour	Dark red	11.6	20	5.8	18.2	8.2	17.6	9.5	19
	Bright red	47.7	50	47.1	63.6	36.1	47.1	45.9	51.7
	Weak red	1.7	-	12.4	9.1	1.6	-	4.7	1.7
	Purple-red	12.4	20	7.4	-	3.3	11.8	9.7	13.8
	Brown-red	7.5	3.3	5.8	-	24.6	17.6	9.5	6.9
	Orange	5.4	-	5.8	9.1	9.8	-	6.1	1.7
	Yellow-brown	2.5	3.3	0.8	-	9.8	-	3.1	1.7
	Yellow	0.8	-	3.3	-	1.6	-	1.7	-
	Brown	-	-	-	-	-	-	0	-
	Grey	5	-	7.4	-	-	-	5	-
	Dark Colours	3.3	3.3	0.8	-	4.9	5.9	2.8	3.4
	Two colours	2.1	-	3.3	-	-	-	2.1	-
Mica	Present	15.4	23.3	38.8	36.4	6.6	5.9	20.8	20.7
Piece size	≤1.5 cm	49	36.7	37.2	27.3	27.9	-	42.6	24.1
	3–1.6 cm	46.5	53.3	51.2	27.3	49.2	52.9	48.2	48.3
	>3 cm	4.6	10	11.6	45.5	23	47.1	9.2	27.6

Values are the percentage frequency of pieces in the unutilized and utilized assemblages, per technocomplex, with each property. An arbitrary cut-off (≥40%) was chosen to highlight elevated values (shaded). A dash (-) represents 0%. The pieces possibly utilized assemblage are excluded here.

Portable XRF was performed on the surface of 129 ochre pieces. Analysis is semi-quantitative and proportions of the major elements in each piece can be obtained. pXRF analysis shows that the utilized pieces, throughout the sequence, have a higher average Fe content compared with the unutilized assemblage, whereas the unutilised pieces have a higher average Si content (Table 5).

**Table 5:** pXRF average percentages of Si and Fe contents in the Rose Cottage Cave ochre pieces.

	Post-HP (n = 69)		HP (n = 40)		Pre-HP (n = 20)		Total (n = 129)	
	Unutilized	Utilized	Unutilized	Utilized	Unutilized	Utilized	Unutilized	Utilized
	n = 57	n = 12	n = 34	n = 6	n = 11	n = 9	n = 102	n = 27
Average % Fe	37.3%	44.6%	29.1%	37.2%	49.2%	50.7%	36.4%	45%
Average % Si	23.9%	17.2%	26.2%	21.5%	21.1%	18.6%	24.1%	18.6%

Few pieces have brilliant or sparkling mica inclusions. Those that do are mostly shales and some are siltstones. More mica-rich pieces are present in the HP than elsewhere. They are particularly uncommon in the pre-HP.

#### Grain size

**Fig 4:**
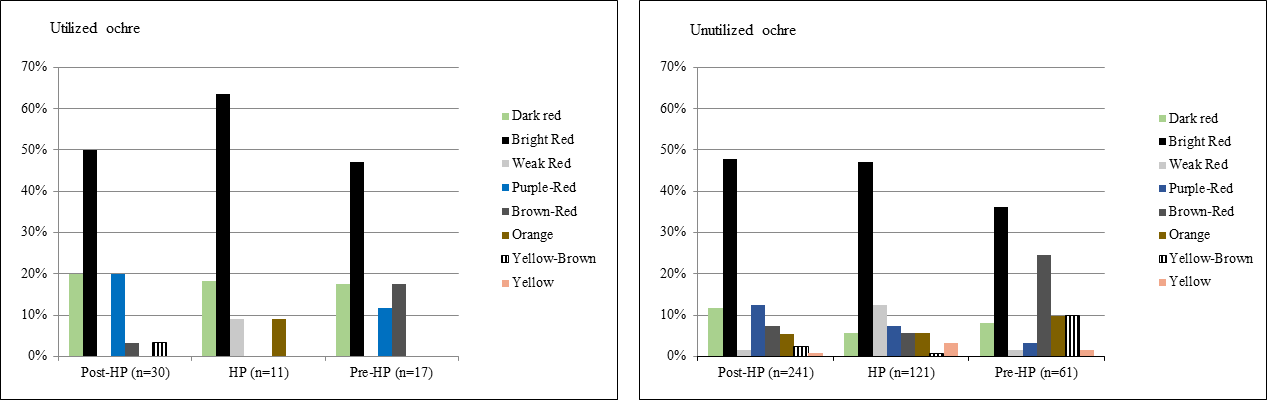
Temporal variations of the percentages of pieces with clayey, silty or sandy grain sizes, in the utilized and unutilized earlier MSA ochre assemblages at Rose Cottage Cave.

Clayey pieces are most common in utilized and unutilized categories, but silty pieces are also well-represented (Table 4). Sandy and silty/sandy pieces are mostly unutilized. Utilized pieces in the pre-HP layers are mostly silty (Fig 4), whereas both the HP and the post-HP have more clayey utilized pieces.

#### Hardness

Soft pieces (Mohs 1 and 2) are common in both the unutilised and utilised assemblages throughout the sequence (Table 4), but the pre-HP layers show an increase of utilised soft pieces compared to the HP and post-HP. Reflecting the geological type preferences in the collection, the utilized and unutilized soft pieces are mostly shales. However, in the post-HP and pre-HP nearly all the utilized soft pieces are shales (94.7% and 83.3% respectively) implying the preferential selection of soft shales for activities.

#### Colour

**Fig 5:**
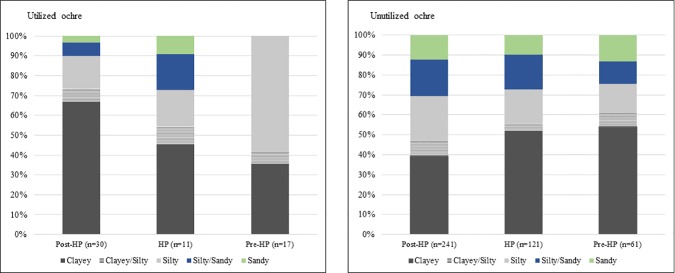
Temporal variations in the frequencies of pieces in the most represented colours groups in the earlier MSA unutilized and utilized ochre assemblages at Rose Cottage Cave.

Colour frequencies of unutilized and utilized ochre pieces are very similar, with bright red dominating both collections (Table 4; Fig 5). Utilized bright-red pieces are especially prevalent in the HP. Dark-reds, purple-reds and brown-reds are present in relatively large numbers, while weak reds and oranges are less frequent, but still common. Pieces with yellow and yellow-brown streaks are scarce, but are found in slightly higher quantities in the unutilized assemblage (Table 4). The unutilized assemblage shows a wider range of colours than the utilized, implying a possible preference to utilize specific colours, namely bright-reds and reds.

#### Ochre piece size

Ochre piece size changes through time (Table 4), with medium sized pieces (3–1.6 cm) common throughout the sequence. The utilized assemblage has a higher percentage of large pieces (maximum length >3 cm) than the unutilized assemblage—27.1% versus 9.8%. Unlike the post-HP pieces, in the pre-HP and HP there are large quantities of utilized large pieces (almost 50% compared to 10% in the post-HP utilized assemblages). There are 89 pieces smaller than 8 mm in the assemblage (these are excluded from the current analysis), but this is a minimum number because small pieces were probably not collected by Malan.

### Utilized ochre

**Fig 6:**
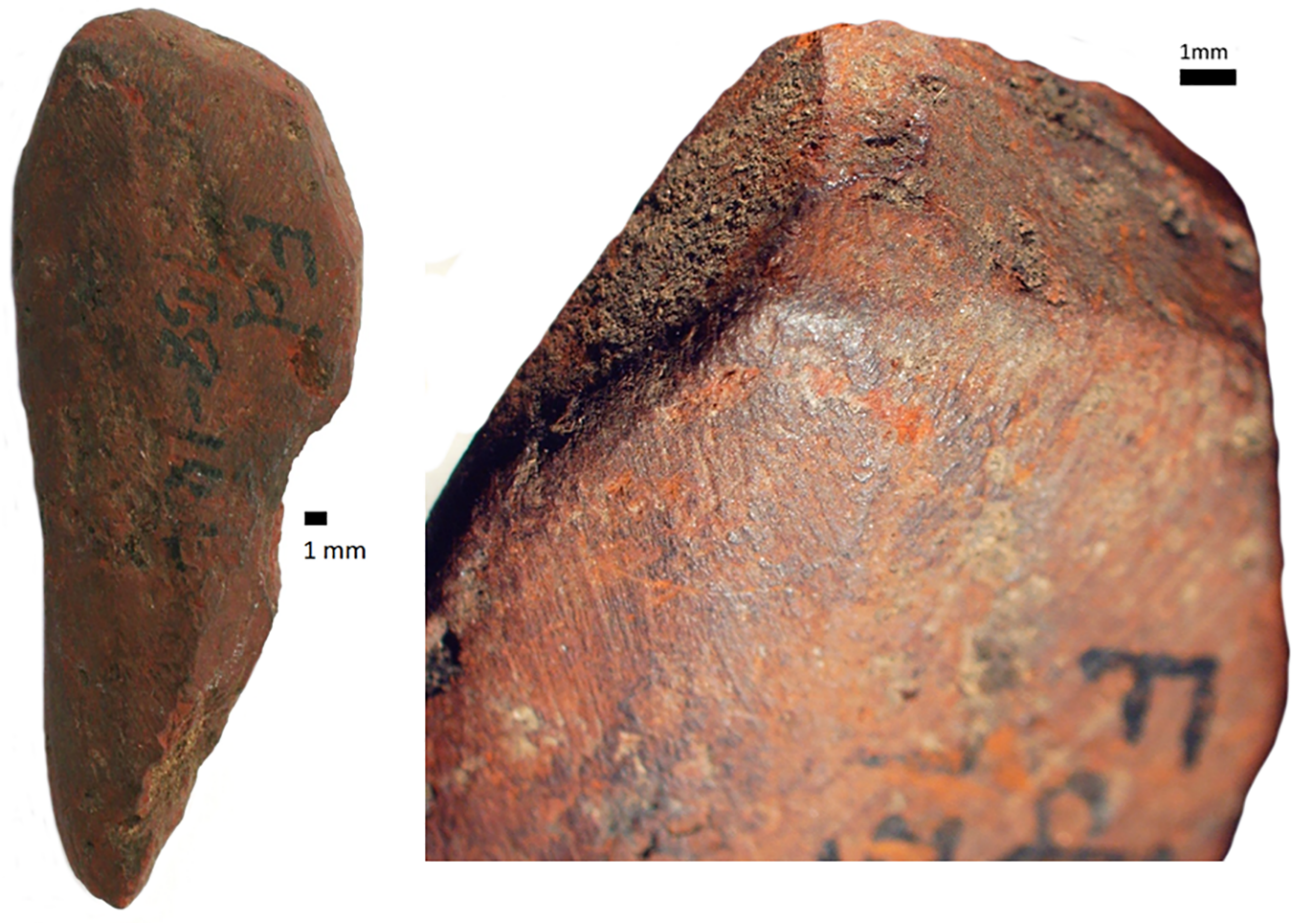
Intensively ground ochre piece from Rose Cottage Cave (RCC-M-079 from post-HP spit 138–144, square Fd). Striated, faceted surfaces and micro-facets are visible around the tip of the piece.

There are 59 pieces with evidence of utilization. Almost 70% of these pieces have utilization on only one or two sides, and 19% have been utilized on four or more sides. Of the intensively utilized pieces, only two are crayon-like, i.e. crayon-shaped pieces that have worked facets on three or more adjacent surfaces and micro-facets on the tip [76] (Fig 6).

Grooves occur on 32 pieces and one quarter of these have microstriations within the grooves. This low number is not only due to secondary activities performed with the ochre (such as rubbing), but is the result of post-depositional processes, handling by researchers and poor curation.

### Ochre processing

Ochre pieces are mainly rubbed, though some are ground, and others are both ground and rubbed. Scoring is extremely rare. We now discuss each of these activity groups.

**Fig 7:**
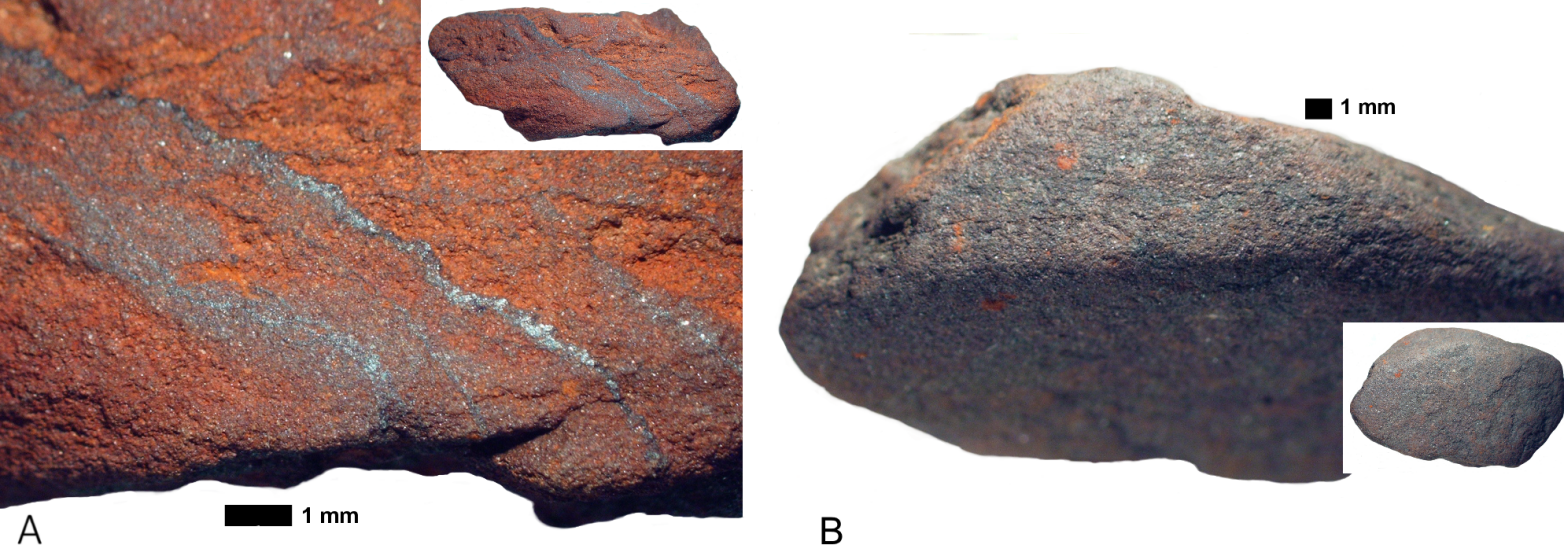
Some ochre pieces from Rose Cottage Cave showing rubbing use-traces. A. Smoothing and metallic lustre, containing external microstriations, on the high ridges on a piece of shale (RCC-H-089 from HP layer SUZ, square Hg). B. Flat surface with rounded edges of a piece of silty, mica-rich shale (RCC-H-263 from post-HP layer JEN, square Ih-Hh).

**Fig 8:**
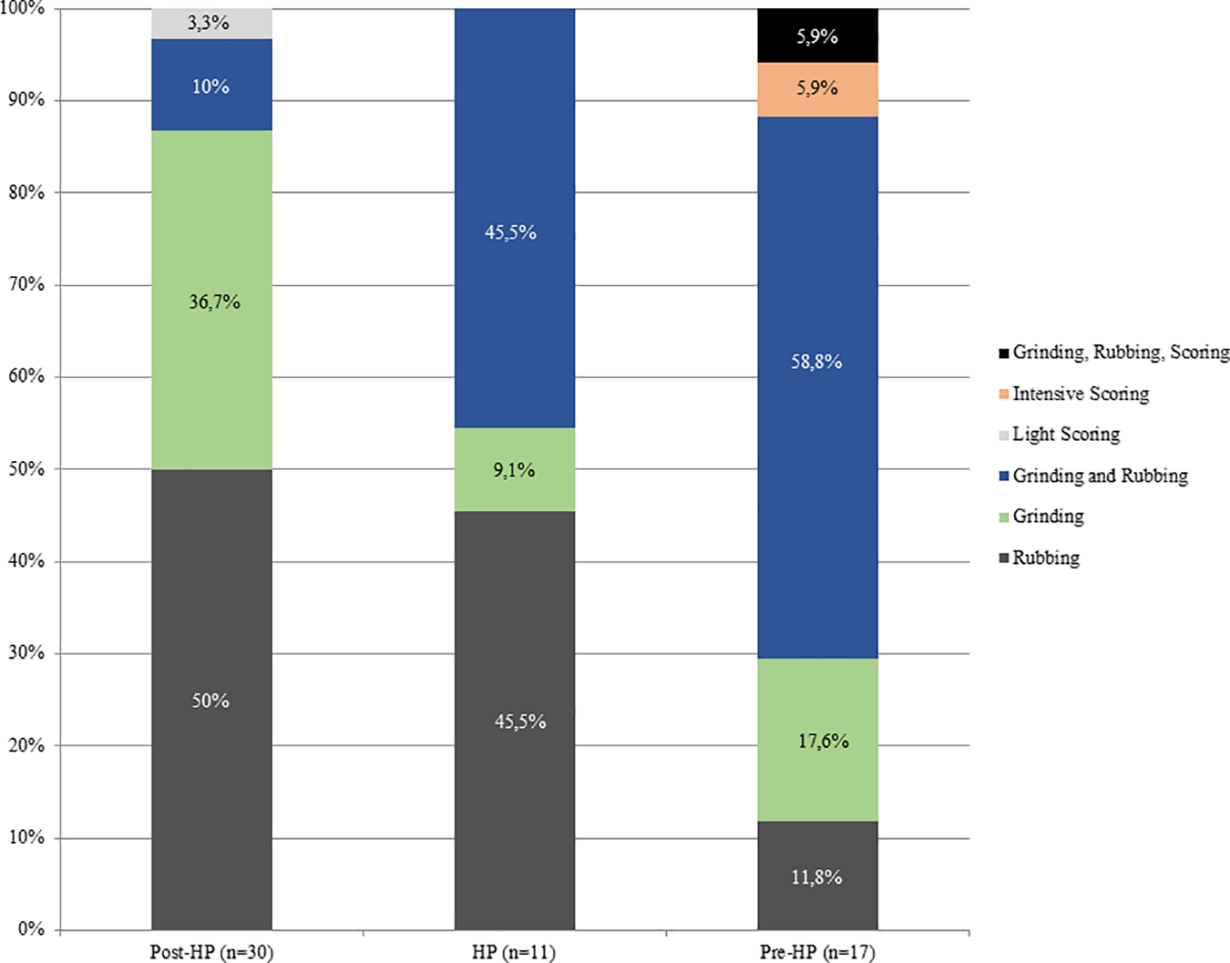
Temporal variations in the frequency of earlier MSA ochre pieces at Rose Cottage Cave used for each activity.

**Fig 9:**
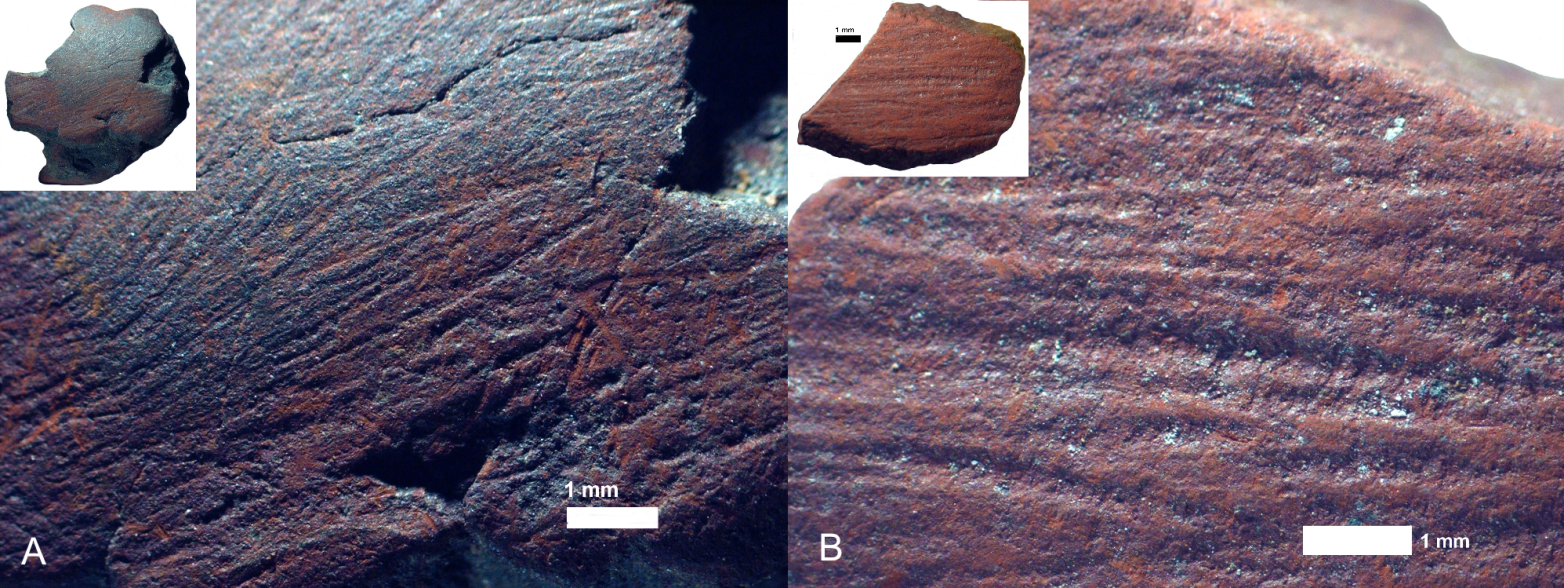
Some ochre pieces from Rose Cottage Cave showing grinding use-traces. A. Parallel and erratic grinding striations on a piece of shale (RCC-H-360 from post-HP layer THO, square Hg). Polish has formed on the striations during the grinding process. B. Wide-U shaped grinding striations, without microstriations on a small shale piece (RCC-H-233 from post-HP layer PAN, square Ig-If). Microstriations were likely removed and striations smoothed during post-depositional processes, since there is no evidence of rubbing use-wear.

**Fig 10:**
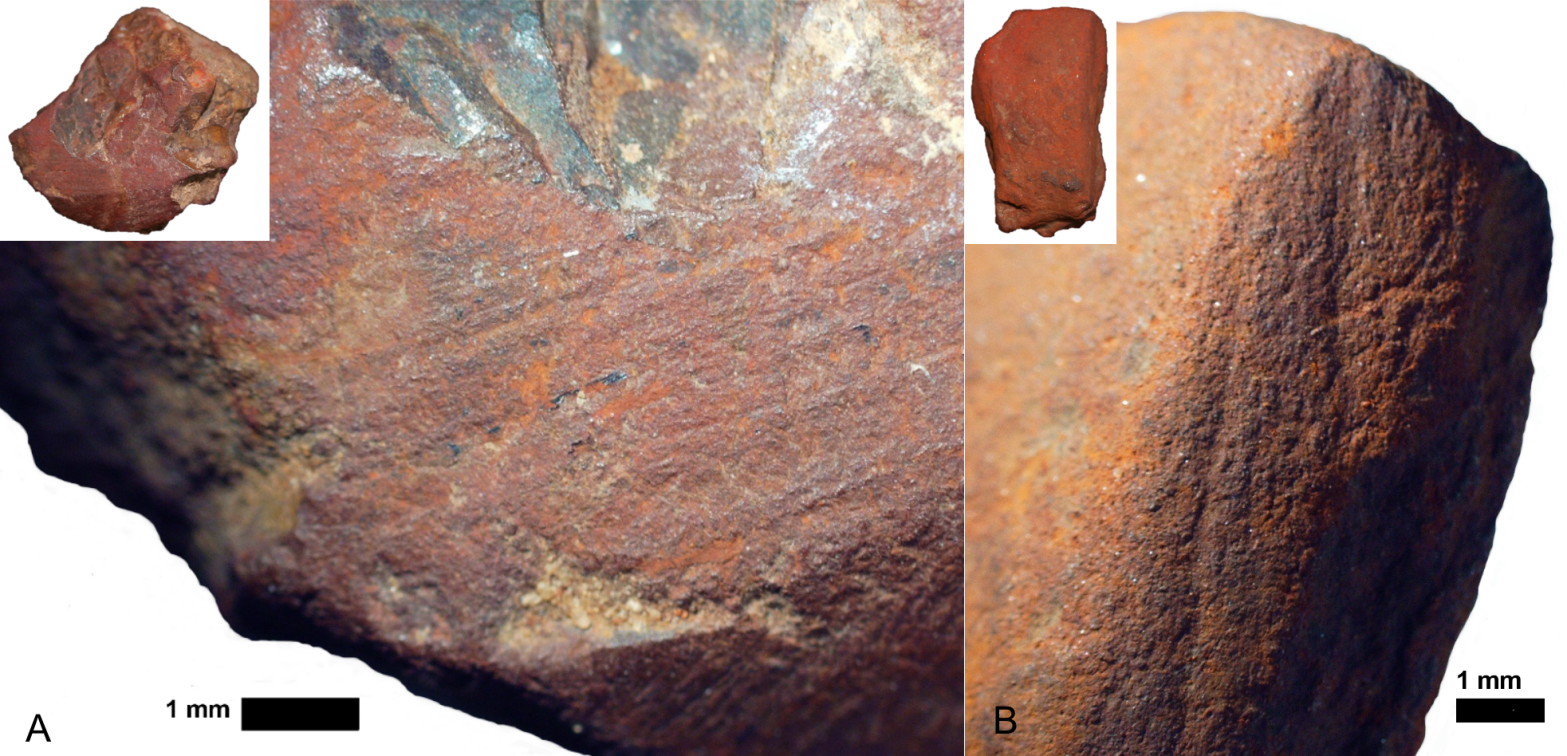
Some ochre pieces from Rose Cottage Cave showing use-traces from combined grinding and rubbing activities. A. Hard iron oxide piece (RCC-M-050 from pre-HP spit 219–225, square Gc) with smoothed grinding striations, and polish and metallic lustre on the high ridges around the striations. B. Smoothed and partially removed grinding striations on a faceted shale piece (RCC-M-069 from post-HP spit 126–132, square Gc). The striations have wide-U profile shapes and no internal microstriations.

**Fig 11:**
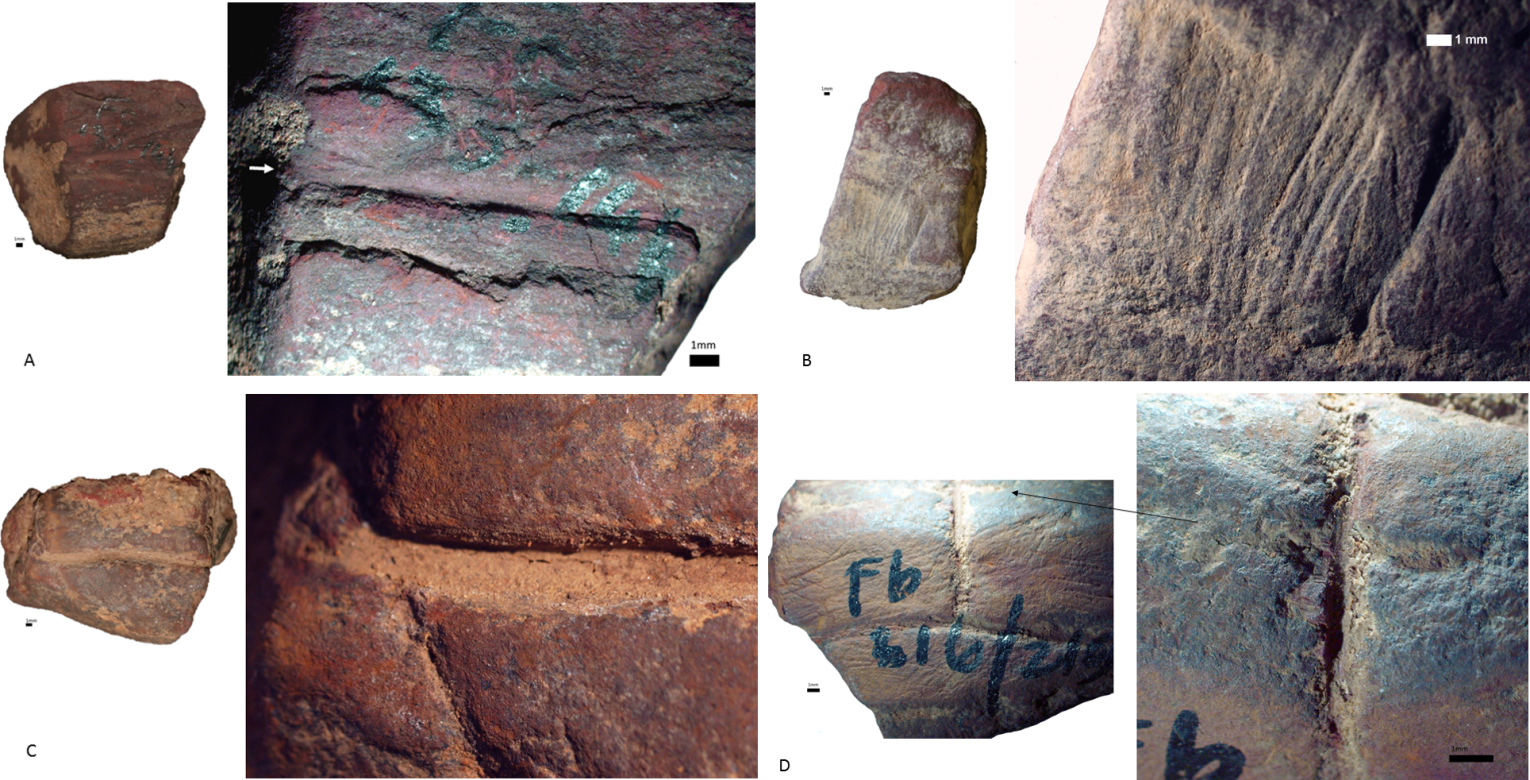
The ochre pieces from Rose Cottage Cave with scoring use-traces. A. Soft shale piece (RCC-M-075 from post-HP spit 133–144, square Fc) with one scored incision (indicated by the arrow) that reaches both edges of the surface. B. Multiple scored, erratically placed striations on a shale piece (RCC-M-019 from pre-HP spit 225–228, square Fb). C. Shale piece (RCC-M-017 from pre-HP spit 216–219, square Fb) bearing deep crossed incisions that have been smoothed. D. The reverse side of RCC-M-017 has grinding and scored incisions that cross over each other.

1. *Rubbing*. There are 23 pieces that are exclusively rubbed. Smoothing is the main indicator of rubbing activities, and it occurs on all the pieces. All, but one, also display external microstriations on the smoothed areas. Polish occurs on 11 pieces and metallic lustre on six (e.g. Fig 7A); with the two use-traces occurring together on two of these pieces. Only three rubbed pieces display grooves, none of which have internal microstriations. Most of the pieces exhibit edge rounding (52.2%) or faceted edges (34.8%), and nearly all pieces have flat utilized surfaces (73.9%) (Fig 7). Surface and edge shape changes occur on many pieces (87%). This high frequency is possibly due to the softness of the ochre pieces, so shape changes could occur after even a short period of rubbing. Pieces with rubbing use-traces frequently have three or more sides used (39.1% versus 20% of ground pieces and 29.4% of combined ground and rubbed pieces), but the same portion (39.1%) of rubbed pieces are utilized on one side. Pieces with use-wear from rubbing are common in the post-HP and HP, but rare in the pre-HP (Fig 8). Most of the rubbed pieces are soft shales, often with a clayey grain size (Table 6). The rubbed pieces predominantly have bright-red or dark red streaks and they have the highest frequency of ochre pieces with mica inclusions, than any other activity categories.
2. *Grinding*. Fifteen pieces were exclusively ground and display parallel striations, sometimes occurring in groups that are at oblique angles to each other, demonstrating a change in the direction of grinding (Fig 9A). Half have wide-U shaped striation profiles and there are no internal microstriations (Fig 9B). Both features are likely to be the result of post-depositional smoothing since there is no other use-trace evidence to suggest that rubbing on a soft material took place after grinding. There is surface shape and edge shape change in all the pieces, with most pieces (73.3%) possessing flat surfaces, with rounded edges or faceted edges. The majority (53.3%) of the ground pieces are utilized on one side only. Grinding is most common in the post-HP and least common in the HP (Fig 8). Two-thirds of the ground pieces have bright-red streaks; therefore most of the grinding activities would have resulted in the production of a bright-red powder (mostly without mica) (Table 6).
3. *Combined grinding and rubbing*. Eighteen pieces display evidence that they were ground and then rubbed (on the same surface). The activities together result in grinding striations that are heavily smoothed or partially removed; the striations no longer have internal microstriations and are wide-U or U-shaped in profile (Fig 10). The pieces often exhibit external microstriations or polish and mostly have flat or convex surfaces (61.1% and 27.8%, respectively), with faceted and rounded edges. Most of these pieces (72%, n = 18) have use-traces on only one or two surfaces. This combined activity occurred mostly in the pre-HP and HP (Fig 8) on soft and medium shales with bright-red streaks (Table 6).
4. *Scoring*. Two pieces display scoring alone (Fig 11A and 11B)–one is lightly and the other intensively scored. The lightly scored piece (Fig 11A) has one re-scored incision, with frayed ends, which appears to have been carefully engraved on the surface. This process would have resulted in the production of minimal amounts of powder. The intensively scored piece has shallow parallel and erratically placed markings (Fig 11B). The scoring on this piece has caused a slightly concave surface shape and would have resulted in powder production.

**Table 6:** Frequencies of the physical properties of the utilized Rose Cottage Cave ochre pieces represented in each activity category of rubbing, grinding, grinding and rubbing, light and intensive scoring.

OCHRE PROPERTIES		Rubbing	Grinding	Grinding and Rubbing	Light scoring	Intensive scoring	Grinding, rubbing and light scoring	Total
		n = 23	n = 15	n = 18	n = 1	n = 1	n = 1	n = 59
		% n	% n	% n	% n	% n	% n	% n
Geological Form	Shale	82.6	60	55.6	100	100	100	69.5
	Mudstone	8.7	6.7	16.7	-	-	-	10.2
	Siltstone	4.3	6.7	5.6	-	-	-	5.1
	Sandstone	-	13.3	5.6	-	-	-	5.1
	Snuffbox Shale	-	-	-	-	-	-	-
	Iron Oxide	4.3	13.3	17	-	-	-	10.2
	Other	-	-	-	-	-	-	-
Grain Size	Clayey	65.2	40	38.9	100	100	100	52.5
	Clayey/Silty	8.7	6.7	5.6	-	-	-	6.8
	Silty	21.7	33.3	44.4	-	-	-	30.5
	Silty/Sandy	4.3	13.3	5.6	-	-	-	6.8
	Sandy	-	6.7	5.6	-	-	-	3.4
Hardness	Soft	82.6	40	61.1	100	100	-	64.4
	Medium	13	53.3	38.9	-	-	100	32.2
	Hard	4.3	6.7	-	-	-	-	3.4
Colour	Dark red	30.4	6.7	11.1	100	-	-	18.6
	Bright red	43.5	66.7	50	-	100	-	50.8
	Weak red	-	-	5.6	-	-	-	1.7
	Purple-red	17.4	20	5.6	-	-	-	13.6
	Brown-red	4.3	-	11.1	-	-	100	6.8
	Orange	-	-	5.6	-	-	-	1.7
	Yellow-brown	-	-	5.6	-	-	-	1.7
	Yellow	-	-	-	-	-	-	-
	Brown	-	-	-	-	-	-	-
	Grey	-	-	-	-	-	-	-
	Dark colours	4.3	6.7	5.6	-	-	-	5.1
Mica	Present	30.4	20	16.7	-	-	-	18.6
Piece Size	≤1.5 cm	43.5	26.7	-	-	-	-	23.7
	3–1.6 cm	52.5	46.7	44.4	100	-	100	49.2
	>3 cm	4.3	26.7	55.6	-	100	-	27.1

Each piece is only represented in one activity category. An arbitrary cut-off (≥30%) was chosen to highlight elevated values (shaded).

One piece has scoring, grinding and rubbing use-traces (Fig 11C and 11D), on multiple sides of the piece. This piece has clear, deep, re-scored incisions on two surfaces–one ground and one unground. The rounding of the edges of the piece and the incisions suggest that rubbing took place after scoring. The base of some of the incisions has a layer of fine clay, masking any microstriations which may be there, but microstriations are visible in some of the other deep incisions. The scoring on this piece mostly seems carefully placed and deliberate, but may have been functional because it would have resulted in considerable powder production.

The intensively scored piece (Fig 11B) has a bright red streak, whereas the lightly scored pieces have dark red and brown-red streaks (Table 6). The scored pieces are mostly soft, clayey shales that are not mica-rich. Two of the scored pieces are from pre-HP layers; one is from the post-HP (Fig 8).

## Discussion

There are clear trends in ochre collection and use in the 60,000 years represented in the Rose Cottage Cave MSA. Bright red ochre derived from soft shale is most common in all the utilized and unutilized assemblages throughout this long period. The unutilized assemblages show a wider range of geological materials, colours, hardness and grain sizes as though, after closer inspection, unsuitable pieces were rejected. Notwithstanding the ubiquitous presence of bright red, soft shale, its treatment was not constant throughout the MSA sequence, indicating that other colours and varieties of ochre were also occasionally sought after.

About 12% of the ochre pieces bear signs of utilization, a figure not unlike that at several other southern African MSA sites. We briefly summarise each of the techniques observed in our study.

### Ochre processing

#### Rubbing

The rubbed pieces are mostly soft, clayey shales, sometimes (particularly in the HP) with shiny mica-inclusions. Rubbing would therefore have resulted in the easy transferal of red (bright and dark red), occasionally shiny, powder directly onto a soft surface such as human skin or animal hide. Smoothing caused by rubbing is more often thought to be associated with ochre grinding tools than with the ochre itself (see, for example, [83]), therefore when ochre is rubbed directly on a surface it is both the product and the tool. Many of the rubbed pieces are small and are often rubbed on four sides, showing high use intensity and suggesting that pieces used for rubbing activities were exhausted (or until they became difficult to hold) before they were discarded.

#### Grinding

Most ground pieces are bright-red with silty or clayey grain sizes. Grinding activities would have produced a bright red powder. However, over half of the ground pieces are only utilized on one side. Although grinding is an efficient way of producing powder, this processing technique may not have been employed when large quantities of ochre were needed. Grindstones are rare and only one has visible ochre staining [75]. Given the softness of most of the pieces at Rose Cottage, one has to consider that pieces may have been crushed to produce ochre powder. This potential technique leaves little or no trace.

A further 18 pieces were used for both grinding and rubbing activities. Some pieces may have needed to be ground to create a powder on the surface of the piece before rubbing them, or to aid the direct transferal of powder onto a soft material, such as skin. However, it is possible that these use-traces were acquired during two unrelated activities at different times. A range of colours were used for these activities, but bright-reds most commonly. The combined ground and rubbed pieces are often large pieces (55.6% are over 3 cm in maximum length). Most of these pieces are utilized on only one or two surfaces, implying that accidental rubbing, such as from transport in a leather pouch [36], is an unlikely cause of the use-wear.

#### Scoring

Pre-HP layers contain two of the three scored pieces found at the site. Although scoring would have produced some ochre powder, this is unlikely to have been the intention because the technique is rare. The apparent intentional placement and orientation of the incisions suggests they likely had significance attached to their creation, and were possibly created with symbolic intent. One MSA burin had ochre on its end suggesting that it may have been used for scoring/engraving ochre [75].

The use-traces recorded on the Rose Cottage ochre imply that it was used for multiple activities. The distinct ochre processing techniques described here imply that specific and different products were the intended outcomes of each.

### What was the ochre used for?

Processing techniques are likely to be proxies for ochre use, but they provide only circumstantial evidence. There is a single ochre use for which we have direct archaeological evidence at Rose Cottage. The HP lithic assemblage contains backed tools, fine bladelets (<25 mm in length) and blades [60, 64]. Ochre traces visible on the backed edges of some HP tools are interpreted as hafting adhesive because there is often a clear line visible between the hafted and unhafted surfaces of the backed tool [45]. Gibson examined 48 backed tools and 28 of these had ochre on their backed edges [45]. The ochre-rich adhesives leave red stains on tool laterals even though the organic component of the adhesive cannot be detected chemically at Rose Cottage because preservation is poor (see [52] supplementary material). We can thus be sure that people at Rose Cottage were making adhesives with ochre powder, as they were at Sibudu (for example, [36, 46, 47]) and at several sites in Africa and elsewhere in the world (for example, [14, 48, 84]).

Experimental manufacture of ochre-loaded adhesive demonstrates the usefulness of ochre powder mixed with other ingredients [35, 36, 42]. Grinding is the best way to produce ochre powder and when this is performed on a rough rock slab, some gritty particles, often quartz grains, become incorporated in the ochre powder [36]. A portion of coarse aggregate is needed for the creation of a successful cement-like product [36] (but contrary results were obtained by [42]). For this reason, the ochre derived from the silty shales used at Rose Cottage would be better for adhesives than the clayey ochre. Curiously, less than 10% of the HP ochre has been ground to produce powder suggesting that small quantities of powder were required, for adhesive production or for other uses. Furthermore, it suggests that the larger quantities of ochre grinding and powder production in the pre- and post-HP, where ochre-loaded adhesive was not observed, must have had another purpose.

At other MSA sites, powdered ochre was sometimes used to make paint that could have been applied to skin or to artefacts. At Blombos, a variety of ingredients, including red ochre powder and an unknown liquid, was mixed in an abalone shell, presumably to create paint [47, 85]. This paint was made about 100,000 years ago, more-or-less at the time of the earliest pre-HP occupation at Rose Cottage. The HP layers in Rose Cottage have the highest percentage of soft, bright-red ochre pieces with mica inclusions and rubbing was a common activity performed at the time, suggesting that the sparkling red colouring was favoured for rubbing directly onto the skin. More recently, contemporary with the post-HP at Rose Cottage, people at Sibudu made paint from a mixture of red ochre and casein (a milk product) [52]. The purpose of the paints at Blombos and Sibudu is unknown, but they may have been used as body paint for ritual purposes or as a lotion for protection against sun or insects.

Assuming that the pieces displaying combined rubbing and grinding use-traces were performed as part of a *chaîne opératoire* of one activity, the ochre might be suitable for creating powder for adhesives, but it seems a better method for colouring and/or tanning hides. When working hides the ochre piece is likely to have been rubbed directly on it, but would occasionally be ground on a stone to remove accumulated fat and rejuvenate the ochre surface for continued direct rubbing. Combined grinding and rubbing could also have been used to create a powdered surface on an ochre piece with which to ‘draw’ a marking on skin.

At Sibudu, preliminary investigation suggests that in the post-HP ochre processing of hides may have been done with scrapers [86]. Hide processing with ochre might also have taken place at Blombos Cave. Polished, ochre-stained bone awls from the site seem to have been used to perforate hides in MSA occupations dated to between 82,000 and 75,000 years ago [19, 87]. Williamson [75] examined Rose Cottage scrapers and did not find ochre on them. Thus, there is no available evidence for ochre tanning or colouring of hides at Rose Cottage. Certainly the ochre would be suitable for the purpose: XRF and pXRF readings on Rose Cottage specimens of ochre pieces demonstrate that the red ochre has a higher average Fe and Fe_2_O_3_ content (Table 5). Red ochre with high iron content is ideal for antibacterial and antifungal hide tanning (see [37, 88]) as well as for other preservative purposes. Rifkin’s [37] hide processing experiments establish that red ochre with high iron content is more effective at preserving hides than yellow ochre or kaolin, and his research supports earlier results obtained by Audouin and Plisson [88]. Rifkin found that it was important to remove fat from the inside of the hide to prevent putrefaction. If this task was performed at Rose Cottage, the slightly abrasive silty ochre that predominates in the pre-HP would be best for rubbing on the hide after initial scraping with stone implements. We accept that we have no secure evidence for hide working at Rose Cottage, but nor do we have secure evidence for the use of the ochre for symbolic purposes.

Hide processing requires space to lay out and peg the hide, and the messy task must be performed away from other activity areas ([89], see photo p 125). The pre-HP utilized ochre that was alternately ground and rubbed was almost entirely recovered from the eastern side of the cave, close to the entrance boulder where light is relatively poor (in Malan’s squares Gb, Gc, Fb and Fc; Fig 1B). The area might have been selected for tasks like hide production that were undertaken irregularly. In contrast, there is only one utilized (rubbed) ochre piece from Harper’s pre-HP assemblage and it was collected closer to the centre of the cave, near the well-lit back wall. Distributions of ochre changed in the post-HP where 22 out of 30 utilized pieces came from Harper’s excavation. This spatial pattern may signify different use of space in the cave through over time.

The post-HP utilized ochre is predominantly clayey and is mostly rubbed, so there are geological and processing differences between the pre- and post-HP assemblages, even though there is some overlap in ochre types and processing methods. Clayey ochre seems more appropriate than silty ochre for rubbing directly on to human skin or pliable objects, or for making compound paint or lotion that could serve the same purpose. High iron oxide content and small grain sizes correlate with superior Sun Protection Factors (SPF), and some ochre that Rifkin and colleagues tested had SPF values greater than 10 thereby showing that ochre can be highly effective as a sunscreen [41]). Experimental and ethnographic studies demonstrate the usefulness of mixing ochre with various substances for use as both sunscreen and mosquito repellent [39–41]. In South Africa, ochre is still used as a skin product, but several purposes are implicated. Red ochre is best known as a skin colouring agent used during ritual activities such as initiations. This ritual use is important, but infrequent. In contrast, many people in KwaZulu-Natal for example use ochre as a sunscreen on their skin on a daily basis. *Ibomvu (*the isiZulu name for kaolin or a clayey colouring agent), is available in most traditional markets. It can be mixed with water and then applied to the skin, but other liquids such as glycerine or commercially available moisturising agents are now sometimes used (Olga Vilane personal communication to LW, 2016). At Sibudu some of the rubbed ochre appears to have been used wet [77, 78] so the practice of applying it with a liquid is likely to be ancient.

## Conclusions

We interpret the time-related differences in ochre products and processing techniques as indicative of changing activities carried out during the MSA of Rose Cottage and we favour the interpretation of multiple uses for ochre at the site. This is an important conclusion that must be considered at other sites, not only in Africa, but elsewhere in the world. The creation and transferal of bright-red and shiny ochre powder was a regular activity at Rose Cottage in the (early) MSA. Ochre is a useful substance for many purposes such as adhesive and sunscreen manufacture and hide tanning, and its colouring potential additionally makes it suitable for symbolic signalling. The presence of rubbed ochre at Rose Cottage, as well as at sites like Sibudu, implies that people sometimes rubbed soft ochre directly on their skin or on pliable objects like hides. While this may have been for practical reasons, symbolic meaning may have been linked to the action, especially since mica-rich, shiny red pieces are preferentially used for this. Further meaning may have been attached to the few engraved pieces found at the site. The purpose or meaning of the engraved pieces is unknown, but the intentional and careful creation of the incisions suggests there was significance attached to their creation.

Rose Cottage Cave has a long MSA sequence spanning more than 60,000 years and change through time in the application of ochre powder is the most likely reason for different geological preferences and processing techniques in the various technocomplexes. Rubbing was the most usual technique of ochre processing in the post-HP, and it was least common in the pre-HP layers. Ochre was ground more often in the post-HP than in the other technocomplexes, but it was never a common technique, and was least common in the HP. Combined grinding and rubbing features prominently in the pre-HP whereas this combined activity was hardly used in the post-HP.

The processing of ochre does not only suggest activities that took place in the past; some of the actions inform us of the cognitive complexity of people at the time. At 96,000 years ago in Rose Cottage, people may have been alternating the techniques of rubbing and grinding with the same pieces of ochre. A complex mental process is involved in rubbing, possibly repeatedly wetting the ochre, then grinding the smoothed surface of the piece on a stone to refresh its ability to transfer ochre powder [90]. The ability to multi-task and switch attention between actions, implies enhanced executive functioning, and which is required for fully exploiting each ochre piece through alternating grinding and rubbing motions [90]. Ochre use in the MSA at Rose Cottage thus provides further evidence of the complex behaviour that was taking place in Africa almost 100,000 years ago.
